# Dabrafenib Treatment in a Patient with an Epithelioid Glioblastoma and BRAF V600E Mutation

**DOI:** 10.3390/ijms19041090

**Published:** 2018-04-05

**Authors:** Garry Ceccon, Jan-Michael Werner, Veronika Dunkl, Caroline Tscherpel, Gabriele Stoffels, Anna Brunn, Martina Deckert, Gereon R. Fink, Norbert Galldiks

**Affiliations:** 1 Department of Neurology, University of Cologne, 50937 Cologne, Germany; garry.ceccon@uk-koeln.de (G.C.); jan-michael.werner@uk-koeln.de (J.-M.W.); veronika.dunkl@uk-koeln.de (V.D.); caroline.tscherpel@uk-koeln.de (C.T.); gereon.fink@uk-koeln.de (G.R.F.); 2 Institute of Neuroscience and Medicine (INM-3, -4), Forschungszentrum Juelich, 52425 Juelich, Germany; g.stoffels@fz-juelich.de; 3 Institute of Neuropathology, University of Cologne, 50937 Cologne, Germany; anna.brunn@uni-koeln.de (A.B.); martina.deckert@uni-koeln.de (M.D.); 4 Center of Integrated Oncology (CIO), Universities of Bonn and Cologne, 50937 Cologne, Germany

**Keywords:** targeted therapy, *BRAF* inhibitors, epithelioid glioblastoma, xanthoastrocytoma

## Abstract

Novel therapeutic targets in malignant glioma patients are urgently needed. Point mutations of the v-Raf murine sarcoma viral oncogene homolog B (*BRAF*) gene occur predominantly in melanoma patients, but may also occur in gliomas. Thus, this is a target of great interest for this group of patients. In a nine-year-old male patient, an anaplastic astrocytoma in the left temporoparietal region was diagnosed histologically. After first- and second-line treatment, a malignant progression to a secondary glioblastoma was observed ten years after the initial diagnosis. Within the following seven years, all other conventional treatment options were exhausted. At this time point, recurrent tumor histology revealed an epithelioid glioblastoma, without a mutation in the isocitrate dehydrogenase gene (*IDH* wild-type). In order to identify a potential target for an experimental salvage therapy, mutational tumor analysis showed a *BRAF V600E* mutation. Consecutively, dabrafenib treatment was initiated. The patient remained clinically stable, and follow-up magnetic resonance images (MRI) were consistent with “Stable Disease” according to the Response Assessment in Neuro-Oncology Working Group (RANO) criteria for the following ten months until tumor progression was detected. The patient died 16 months after dabrafenib treatment initiation. Particularly in younger glioma patients as well as in patients with an epithelioid glioblastoma, screening for a *V600E BRAF* mutation is promising since, in these cases, targeted therapy with *BRAF* inhibitors seems to be a useful salvage treatment option.

## 1. Introduction

Glioblastoma is the most common and aggressive form of brain tumor, with a median survival of only 15–20 months despite maximal multimodal therapy [1,2,3]. Therefore, the search for novel therapeutic targets in these tumors is warranted.

Over a decade ago, systematic genome-wide screening analyses revealed that somatic point mutations activate the v-Raf murine sarcoma viral oncogene homolog B (*BRAF*) kinase and may constitute a target for new therapeutic opportunities in malignant melanoma and other forms of cancer [4]. The *V600E* mutation is found in approximately two-thirds of patients with malignant melanoma [4,5]. In brain tumors, a similar occurrence was described in both pleomorphic and anaplastic pleomorphic xanthoastrocytomas, while it was less commonly found in gangliogliomas (approximately 20%) and pilocytic astrocytomas (approximately 10%) [6]. In contrast, the occurrence of the *V600E* mutation in glioblastoma patients is rare. In a publication by Basto and colleagues, it was found in 2 out of 34 (6%) glioblastoma patients [7].

The introduction of *BRAF* inhibitors targeting the *V600E* mutation such as dabrafenib and vemurafenib represented a treatment breakthrough for patients with malignant melanoma. Currently, in these patients, *BRAF* inhibition is the treatment of choice if the *V600E* mutation is present [8,9]. While there is also evidence for the efficacy of these substances in patients with non-small cell lung cancer [10], data on the use of *BRAF* inhibitors in patients with malignant glioma are scarce [11,12,13,14,15].

Remarkably, our patient presented with an epithelioid glioblastoma, a variant characterized by large epithelioid melanoma-like cells, comparatively young age of onset, the presence of a *BRAF V600E* mutation in approximately 50% of cases, and absence of a mutation in the isocitrate dehydrogenase gene (*IDH* wild-type) [16,17,18,19,20,21].

We here present a young patient with an *IDH* wild-type epithelioid glioblastoma exhibiting a *V600E* point mutation of the *BRAF* gene, in whom clinical and radiological stability could be achieved for ten months by *BRAF* inhibition using dabrafenib as salvage therapy.

## 2. Case Description

**Figure 1:**
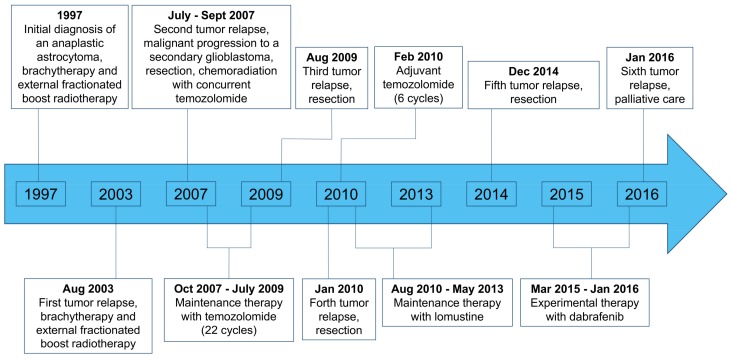
Overview of the patient’s course of disease and treatment regimens.

At the age of 9 years, an anaplastic astrocytoma (grade III according to the World Health Organization (WHO) classification of the central nervous system) in the left temporoparietal region of a male patient was diagnosed histologically. First-line therapy consisted of interstitial brachytherapy using ^125^I-seeds and external boost radiotherapy. During the further course of the disease over many years, multiple tumor relapses occurred and numerous treatment options were used. A detailed treatment overview is depicted in Figure 1.

In 2007, i.e., ten years after the initial diagnosis, a malignant progression to a secondary glioblastoma (WHO grade IV) was diagnosed (Figure 1). Since all other conventional treatment options had been exhausted, and to find a target for an experimental salvage therapy, recurrent tumor tissue was obtained via surgery in 2014 and molecularly analyzed. Histology was consistent with an *IDH* wild-type epithelioid glioblastoma and the mutational analysis revealed a *V600E* mutation of the *BRAF* kinase. Consecutively, dabrafenib therapy was initiated (150 mg twice daily).

**Figure 2:**
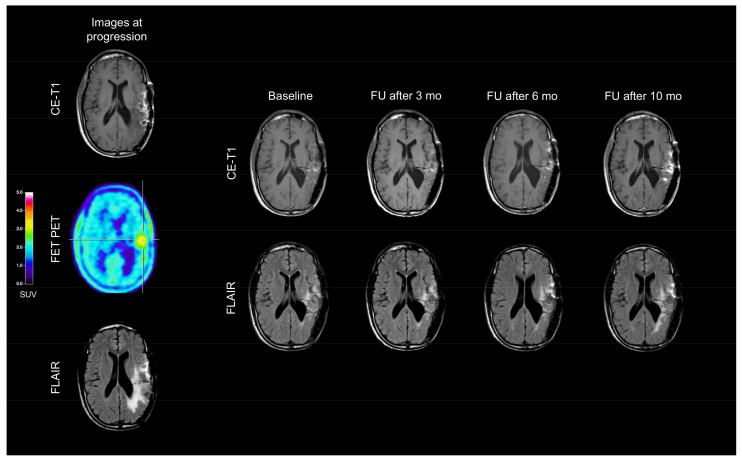
In December 2014, Magnetic Resonance Imaging (MRI; left column) shows a contrast-enhancing lesion and an enlarged FLAIR hyperintensity in the left temporoparietal lobe. The corresponding Positron-Emission-Tomography (PET) scan using *O*-(2-^18^F-fluoroethyl)-l-tyrosine (FET) depicts increased metabolic activity in spatial correlation with the contrast enhancement. MR and PET images are consistent with tumor progression. Compared to baseline MRI, follow-up (FU) MRI findings (right rows) during dabrafenib therapy remain unchanged until the tumor progression ten months after dabrafenib initiation.

Following dabrafenib, the clinical follow-up was stable, and serial magnetic resonance imaging (MRI) scans revealed no further tumor progression (“Stable Disease” according to the Response Assessment in Neuro-Oncology Working Group (RANO) criteria) for ten months (Figure 2). Ten months after dabrafenib treatment initiation, MRI exhibited tumor progression, and dabrafenib therapy was discontinued (Figure 2). The patient requested no further oncological treatment and died six months later.

## 3. Discussion

In our patient with an *IDH* wild-type epithelioid glioblastoma and a V600E mutation of the *BRAF* kinase treated with dabrafenib as salvage therapy, we achieved clinical and radiological stability over ten months, which is remarkable at that point of the clinical course with extensive pretreatment (Figure 1). Thus, targeted therapy with *BRAF* inhibitors may constitute a valuable salvage treatment option. Furthermore, this case suggests that in selected patients, i.e., in younger and heavily pretreated patients without further conventional treatment options, it may be helpful to assess whether a *BRAF* mutation, especially if an epithelioid glioblastoma, is present.

In malignant melanoma patients with a *V600E* point mutation of the BRAF gene, targeted therapy using *BRAF* kinase inhibitors has dramatically improved the prognosis [8,9]. In contrast, data about its efficacy in patients with brain tumors, and notably in glioblastomas, are scarce. Targeted therapies such as vemurafenib or dabrafenib have been used only in a limited number of brain tumor patients with predominantly pleomorphic xanthoastrocytoma and ganglioglioma [11,12,14,15]. Meletath and co-workers reported that dabrafenib in combination with tumor-treating fields yielded a remarkable clinical and radiologic response over 24 months in a patient with a recurrent malignant glioma arising from ganglioglioma [12]. Chamberlain [15] treated three adult patients with refractory ganglioglioma and *BRAF V600E* mutation with dabrafenib. The median progression-free survival was seven months (range: 4–10 months). In another case series by the same author [14], similar results were observed in four patients with *BRAF V600E*-mutated and recurrent pleomorphic xanthoastrocytoma treated with vemurafenib. In a recently published case series, Burger and colleagues [13] reported an impressive radiological response and a stable clinical course up to 27 months in patients with malignant *BRAF V600E*-mutated glioma and leptomeningeal tumor manifestation using dabrafenib monotherapy. In this case series, histology revealed a glioblastoma in one of the three cases, while the other diagnoses were consistent with anaplastic pleomorphic xanthoastrocytomas.

**Figure 3:**
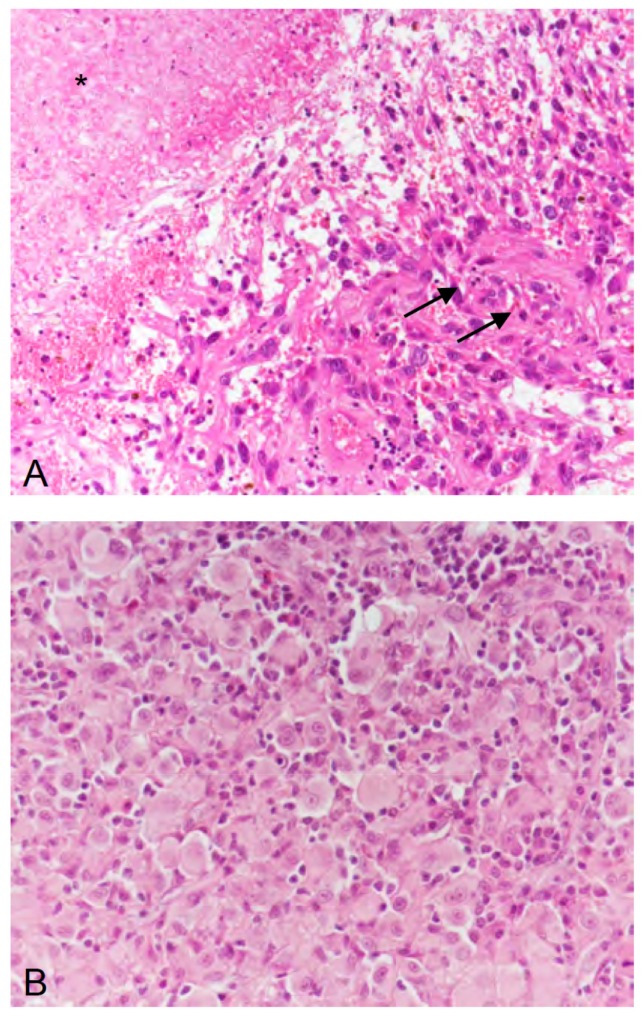
(**A**) In 2007, the second recurrence of the tumor exhibited microvascular proliferation (arrows) and necrosis (asterisk), thus, corresponding to a glioblastoma (WHO grade IV). (**B**) In 2014, the fifth recurrence of the tumor was dominated by epithelioid differentiated glial tumor cells, thus, corresponding, to epithelioid glioblastoma (WHO grade IV). (**A**,**B**) hematoxylin and eosin staining; original magnification ×400.

By reviewing our patient’s history from 1997 to 2016, it can be discussed whether, in contrast to the initial diagnosis (anaplastic astrocytoma with subsequent malignant progression to a secondary glioblastoma), another histological entity such as anaplastic pleomorphic xanthoastrocytoma was initially present. Indeed, in one of the cases reported by Burger and co-workers [13], a re-evaluation of histology by an external reference neuropathologist revealed an anaplastic pleomorphic xanthoastrocytoma, although initially a glioblastoma had been diagnosed. Note that a close relationship between anaplastic pleomorphic xanthoastrocytoma and epithelioid glioblastoma, a glioblastoma subtype, has been discussed previously [22,23]. However, in our case, we have also discussed whether the primary tumor of our patient may correspond to a pleomorphic xanthoastrocytoma, which, however, was excluded by a reference neuropathologist when (in 2007) malignant progression to a glioblastoma occurred. Remarkably, though, while preparing this case report, the retrospective neuropathological evaluation of tissue obtained before dabrafenib therapy in 2014 revealed an epitheloid glioblastoma (Figure 3). This entity has recently been introduced into the updated WHO classification for brain tumors in 2016 [21]. In this rare glioblastoma subtype, a *BRAF V600E* mutation can be detected in approx. 50% of the cases [16,17,18]. Furthermore, the early manifestation (i.e., the onset of the brain tumor at the age of 9 years) is rather typical for patients with *BRAF*-mutated malignant glioma [24] including epithelioid glioblastoma [18].

The survival of our patient is comparable with that of other patients exhibiting a BRAF V600E mutation [17,25,26]. A recent meta-analysis in glioma patients demonstrated an improved overall survival (hazard ratio: 0.6) if a *BRAF* mutation was present [27]. That meta-analysis also revealed that a *BRAF V600E* mutation improved the survival of children and young adults (i.e., under 35 years) with gliomas but did not have prognostic value in older adults. On the other hand, children with a newly diagnosed epithelioid glioblastoma suffer from an overall poor prognosis, independent of a *BRAF V600E* mutation [18]. Furthermore, as reported by Kanamori and colleagues, it is tempting to speculate whether a *BRAF V600E* mutation may be a driver mutation for malignant transformation in an epithelioid glioblastoma [28]. On the other hand, Kuroda et al. described a case with a discrepancy in the *BRAF V600E* mutation states between epithelioid glioblastoma and a colocalized low-grade astrocytoma [29], indicating that an epitheloid glioblastoma may also occur without a *BRAF V600E* driver mutation.

Regarding further treatment options for tumor patients with a *BRAF V600E* mutation, various studies have suggested that, in patients with malignant melanoma, selective *MAPK* kinase (*MEK*) inhibitors such as trametinib in combination with *BRAF* inhibitors are also highly active [30]. A phase 3 trial showed an improvement in overall and progression-free survival of combined targeting of *MEK* and *BRAF* versus *BRAF*-inhibition alone for the first-line treatment of *BRAF V600*-mutated patients with metastatic melanoma [31]. A more recent phase 3 trial showed that the adjuvant therapy of *MEK*- plus *BRAF*-inhibitors showed a lower risk of recurrence following resection of stage III *BRAF V600* mutated melanoma [32]. Thus, it is reasonable to use a combined *BRAF* and *MEK* blockade for the treatment of malignant gliomas with the *V600E* mutation. Efficacy of this combinational approach has already been described in in-vitro and animal studies [33], and also in a patient with a relapsed anaplastic pleomorphic xanthoastrocytoma. In that patient, clinical stability was obtained by *BRAF/MEK* double blockade for at least 11 months [34].

However, to the best of our knowledge, no reports exist of non-responding patients, even if a *BRAF* mutation is present. Future studies with a higher number of patients are warranted to confirm these preliminary but promising results. Furthermore, it has been demonstrated that vemurafenib and dabrafenib have a limited ability to cross the blood–brain barrier [35]. Thus, a future effort should be directed to develop new *BRAF* inhibitors that can cross the blood–brain barrier.

In summary, particularly in younger glioma patients and in patients with an epithelioid glioblastoma, screening for the *V600E* mutation of the *BRAF* gene appears to be promising, since in these cases targeted therapy with *BRAF* inhibitors seems to be a valuable salvage treatment option.
